# Suppressive effect of syndecan ectodomains and *N*-desulfated heparins on osteoclastogenesis via direct binding to macrophage-colony stimulating factor

**DOI:** 10.1038/s41419-018-1167-8

**Published:** 2018-11-02

**Authors:** Jin-Man Kim, Kyunghee Lee, Mi Yeong Kim, Hong-In Shin, Daewon Jeong

**Affiliations:** 1 Department of Microbiology, Laboratory of Bone Metabolism and Control, Yeungnam University College of Medicine, Daegu, 42415 Korea; 2 Asan Medical Center, Asan Institute for Life Sciences, Seoul, 26493 Korea; 3 Department of Oral Pathology, Institute for Hard Tissue and Bio-Tooth Regeneration, School of Dentistry, Kyungpook National University, Daegu, 41940 Korea

## Abstract

Syndecans, a family of cell surface heparan sulfate proteoglycans, regulate cell differentiation via binding of their heparan sulfate chains to growth factors and cytokines and play a role in tumor growth and progression, wound repair, and intestinal mucosal damage. However, the functional and mechanistic roles of syndecans in osteoclast differentiation and bone metabolism are yet unclear. Here, we demonstrated that post-translationally glycosylated ectodomains of syndecan-1 to 4 obtained from mammalian cells efficiently suppressed osteoclast differentiation compared to those obtained from *Escherichia coli* with no systems for glycosylation. A concomitant decrease in the expression of osteoclast markers such as nuclear factor of activated T cells 1 (NFATc1), c-Fos, and ATP6V0D2 was observed. In addition, heparan sulfate and selectively *N*-desulfated heparin derivatives with 2-O- and 6-O-sulfate groups and no anticoagulant activity in blood inhibited osteoclast differentiation. The inhibitory effects of syndecan ectodomains, heparan sulfate, and *N*-desulfated heparin derivatives on osteoclast differentiation were attributed to their direct binding to the macrophage-colony stimulating factor (M-CSF), resulting in the blocking of M-CSF-mediated downstream signals such as extracellular signal-regulated kinase (ERK), c-JUN N-terminal kinase (JNK), p38, and Akt. Furthermore, mice injected with syndecan ectodomains, heparan sulfate, and *N*-desulfated heparin derivatives into periosteal regions of calvaria showed reduction in the formation of tartrate-resistant acid phosphatase (TRAP)-positive mature osteoclasts on the calvarial bone surface, thereby exhibiting decreased bone resorption. Together, these results revealed a novel role of heparan sulfate chains of syndecan ectodomains in the regulation of osteoclast differentiation.

## Introduction

Bone formation and remodeling are regulated by the complex interplay between osteoblasts and osteoclasts in the bone marrow environment. The excessive bone resorption by osteoclasts relative to bone formation by osteoblasts leads to pathologic bone loss such as in osteoporosis, periodontal disease, cancer-associated bone disease, rheumatoid arthritis, and Paget’s disease^1^. Osteoclastogenesis is primarily regulated by two cytokines, macrophage colony-stimulating factor (M-CSF) and receptor activator of nuclear κB ligand (RANKL), which are expressed and released by osteoblasts in the bone marrow^2,3^. M-CSF functions in the proliferation and differentiation of osteoclast precursors by binding to its receptor c-Fms localized on the cytoplasmic membrane of osteoclast precursors and subsequently stimulating diverse signal transduction pathways, including Akt and mitogen-activated protein kinases (MAPKs) such as extracellular signal-regulated kinase (ERK), c-JUN N-terminal kinase (JNK), and p38^4^. RANKL promotes mononuclear osteoclast precursor fusion, followed by multinucleated osteoclast maturation^3,5^.

Proteoglycans comprise a protein core that may covalently attach to at least one glycosaminoglycan (GAG) chain at specific sites^6^. GAGs are unbranched polysaccharides with a backbone of repeating disaccharide units and classified into four groups, namely, heparin/heparan sulfate, chondroitin sulfate/dermatan sulfate, keratan sulfate, and hyaluronic acid^7^. Among various constituents of proteoglycans, heparan sulfate proteoglycans, which play an essential role in embryological development and normal physiology, have enormous structural diversities in heparan sulfate chains in terms of chain length and size, saccharide composition and arrangement, and sulfation and epimerization pattern within the sugar segments^8,9^. Distinct structures of heparan sulfate chains in the same core protein are produced in different cell types and at different tissue sites^10^. Heparan sulfate chains have been found to bind to a wide range of cellular components such as fibroblast growth factors (FGFs) and their receptors, transforming growth factors, interleukins, bone morphogenetic proteins, lipases and apolipoproteins, and extracellular matrix proteins^9,11^.

Syndecans, a family of heparan sulfate proteoglycans that are represented by four members having similar structural organization (syndecan-1 to 4) in mammals, have been reported to regulate cell adhesion, spreading, migration, proliferation, survival, and differentiation as well as the maintenance of cell morphology^12,13^. Syndecan-1 and syndecan-3 are mainly expressed in the epithelial and neuronal cells, respectively^12,14^. Syndecan-2 is mostly present in mesenchymal and smooth muscle cells and syndecan-4 is ubiquitously expressed^15^. Syndecans comprise three distinct domains, including a C-terminal cytoplasmic domain, transmembrane domain, and N-terminal extracellular domain (ectodomain). The ectodomain of syndecans contains heparan sulfate and chondroitin sulfate GAG side chains and exhibits the ability to bind with growth factors and cytokines such as FGF-2, hepatocyte growth factor (HGF), and interleukin-8^16–19^. All syndecans are shown to carry heparan sulfate chains, and syndecan-1 and syndecan-3 display both heparan sulfate and chondroitin sulfate chains in the ectodomain^20^. Extracellular shedding of syndecans to generate soluble ectodomains has been reported to constitutively occur under physiological conditions or may be significantly induced by stimuli-mediated activation of various proteases such as matrix metalloproteinase-2 (MMP-2), MMP-7, MMP-9, and membrane-associated MT1-MMP and MT3-MMP^21–23^. Shed ectodomains of syndecans were shown to play an important role in pathophysiological events such as tumor growth and progression, wound repair, and intestinal mucosal damage^19,24,25^. Syndecan-1 ectodomains from myeloma cell lines were shown to inhibit osteoclast formation and induce osteoblast development in bone marrow cell cultures^26^, although the detail mechanisms remain largely unknown.

In this study, we identified that syndecan ectodomains and heparan sulfate chains negatively regulate osteoclast differentiation via direct interaction with M-CSF, as evident from a pull-down, an immunosorbent-based, and a competitive binding assay. In addition, selectively *N*-desulfated heparin derivatives with no anticoagulant activity suppressed osteoclastogenesis. Consistent with the results of in vitro studies, the subcutaneous administration of syndecan ectodomains, heparan sulfate, and *N*-desulfated heparin derivatives into mouse calvaria resulted in a decrease in osteoclast formation and bone resorption on the surface of the calvarial bone. From these results, we elucidated the functional mechanism underlying the inhibitory action of heparan sulfate of syndecan ectodomains on osteoclast differentiation.

## Results

### Syndecan ectodomains suppress osteoclast differentiation

**Fig. 1:**
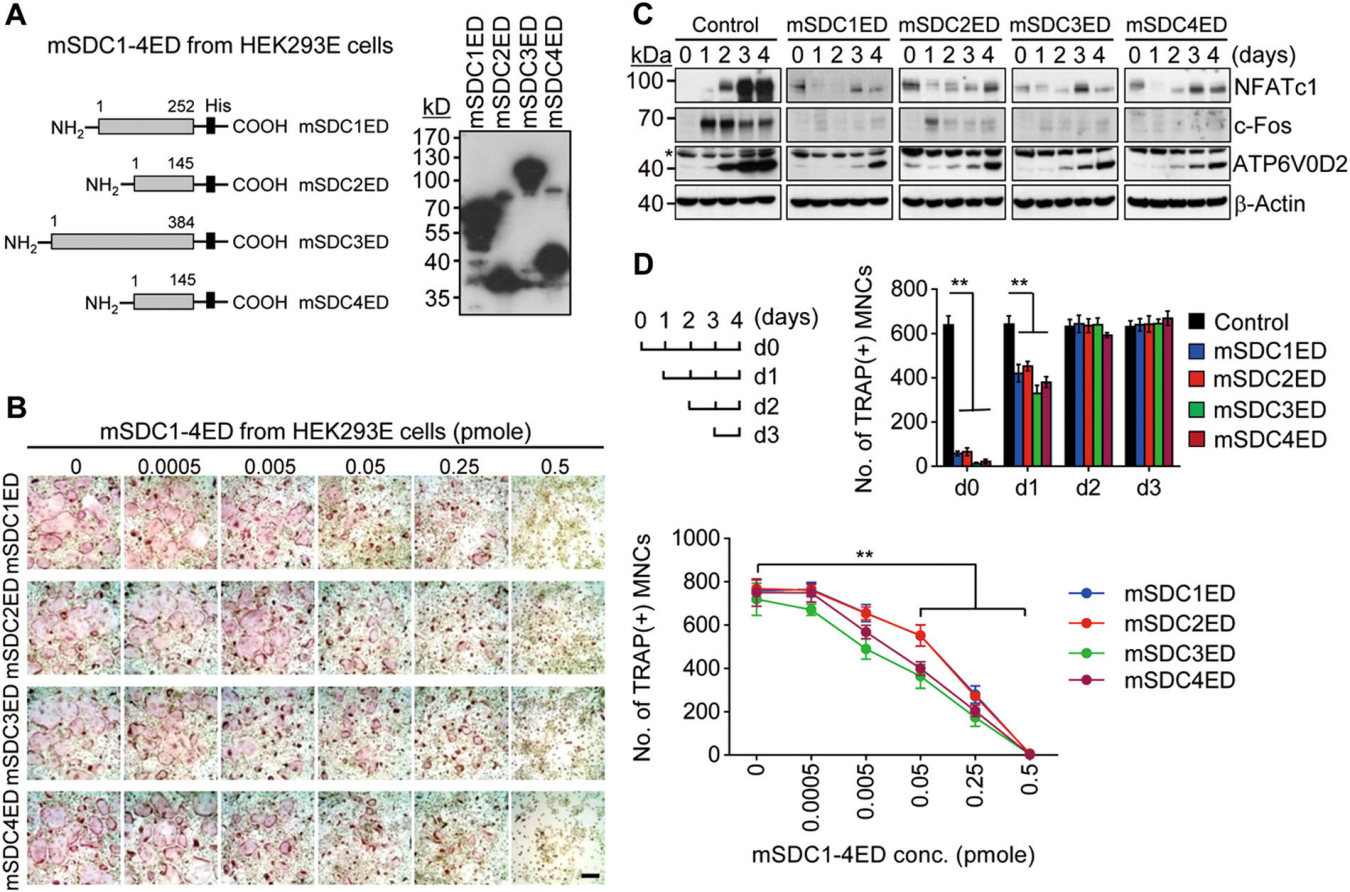
Ectodomains of syndecan-1 to 4 from HEK293E cells block osteoclast differentiation. **a** Schematic diagram of mouse syndecan-1 to 4 ectodomains (mSDC1ED to mSDC4ED) expressed from HEK293E cells. The gray and black boxes represent the syndecan ectodomain and histidine (His), respectively (left panel). The recombinant syndecan ectodomains purified from HEK293E cells were separated by electrophoresis on a 10% SDS-PAGE and immunoblotted with anti-His antibody (right panel). The positions of the molecular mass markers are represented on the left. **b** Inhibitory effect of syndecan ectodomains on osteoclast differentiation. Bone marrow-derived osteoclast precursors were treated with various concentrations of recombinant syndecan ectodomains in the presence of M-CSF (30 ng/mL) and RANKL (100 ng/mL) for 4 days. Cells were fixed and stained for TRAP. The number of TRAP-positive osteoclasts [TRAP(+) MNCs] was counted under a light microscope. Scale bar, 200 μm. **c** Osteoclastogenic marker genes. Osteoclast precursors were treated with syndecan ectodomains (1 nM) in the presence of M-CSF and RANKL for 4 days. Cell lysates were subjected to immunoblot analysis using antibodies specific for NFATc1, c-Fos, and ATP6V0D2. β-Actin was used as a loading control. Asterisk indicates non-specific bands. **d** Osteoclast precursors were treated with syndecan ectodomains (1 nM) at the indicated time points during osteoclast differentiation. After TRAP staining, the number of TRAP(+) MNCs was counted. Results represent the means ± SD (*n* *=* 3). **p *<* 0.01

The ectodomains of mouse syndecan-1 to 4 (mSDC1ED to mSDC4ED) were expressed in two different cell types, mammalian HEK293E cells and *E. coli* Rosetta 2 (DE3) strain. The corresponding amino acids of syndecan ectodomains from HEK293E cells and *E. coli* are presented in Fig. 1a and Supplementary Fig. 1a (left panel), respectively. The molecular weights of ectodomains of syndecan-1, 2, 3, and 4 from HEK293E cells were approximately 70, 37.5, 120, and 40 kDa, respectively, and those of ectodomains of syndecan-1, 2, 3, and 4 from *E. coli* were approximately 42, 25, 70, and 30 kDa, respectively (Fig. 1a and Supplementary Fig. 1a; right panel). Despite the similarity of predicted molecular weights of syndecans expressed in two different cell expression systems, the molecular weights of syndecan ectodomains from HEK293E cells were much higher than those of ectodomains from *E. coli*, suggesting that syndecan ectodomains expressed in mammalian cells may carry heparan sulfate and chondroitin sulfate side chains. These observations are consistent with previous reports, wherein syndecan-bearing heparan sulfate and chondroitin sulfate chains exhibited higher molecular weights than their expected sizes^26,27^.

We next examined the effect of syndecan ectodomains on the differentiation of bone marrow-derived osteoclast precursors into osteoclasts in the presence of M-CSF and RANKL. Recombinant ectodomains of syndecan-1 to 4 from HEK293E cells and *E. coli* inhibited osteoclast differentiation in a dose-dependent manner (Fig. 1b and Supplementary Fig. 1b), with a simultaneous decrease in the expression level of osteoclastogenic marker genes such as nuclear factor of activated T cells 1 (NFATc1), c-Fos, and ATP6V0D2 (Fig. 1c). The maximum inhibitory dose of syndecan ectodomains from HEK293E cells was 1 nM, whereas bacterially produced syndecan ectodomains lacking GAG side chains failed to block osteoclast differentiation at the same concentration. This observation suggests that heparan/chondroitin sulfate chains of syndecan ectodomains may play a critical role in osteoclastogenesis. On the contrary, syndecan ectodomains from *E. coli* suppressed osteoclast differentiation at concentrations ranging from 100 to 6000 nM (Supplementary Fig. 1b), indicating that much higher concentrations of core proteins of syndecan ectodomains may contribute to the regulation of osteoclast differentiation in a different way from GAG side chains. To determine the time point of the inhibitory action of syndecan ectodomains during the multi-step process of osteoclast differentiation, cells were treated with syndecans at different time points post-differentiation. The inhibitory effect was effective when syndecans were treated at the early stage (day 0 to 1) of osteoclast differentiation, whereas such a phenomenon disappeared with the progression of differentiation (Fig. 1d). In addition, we observed that syndecans blocked the activity of tartrate-resistant acid phosphatase (TRAP), an early marker of osteoclastogenesis, in a dose-dependent manner (Supplementary Fig. 2). Taken together, syndecan ectodomains produced in mammalian cells may suppress the early stage of osteoclastogenesis.

### Syndecan ectodomains inhibit osteoclastogenesis through direct interaction with M-CSF

**Fig. 2:**
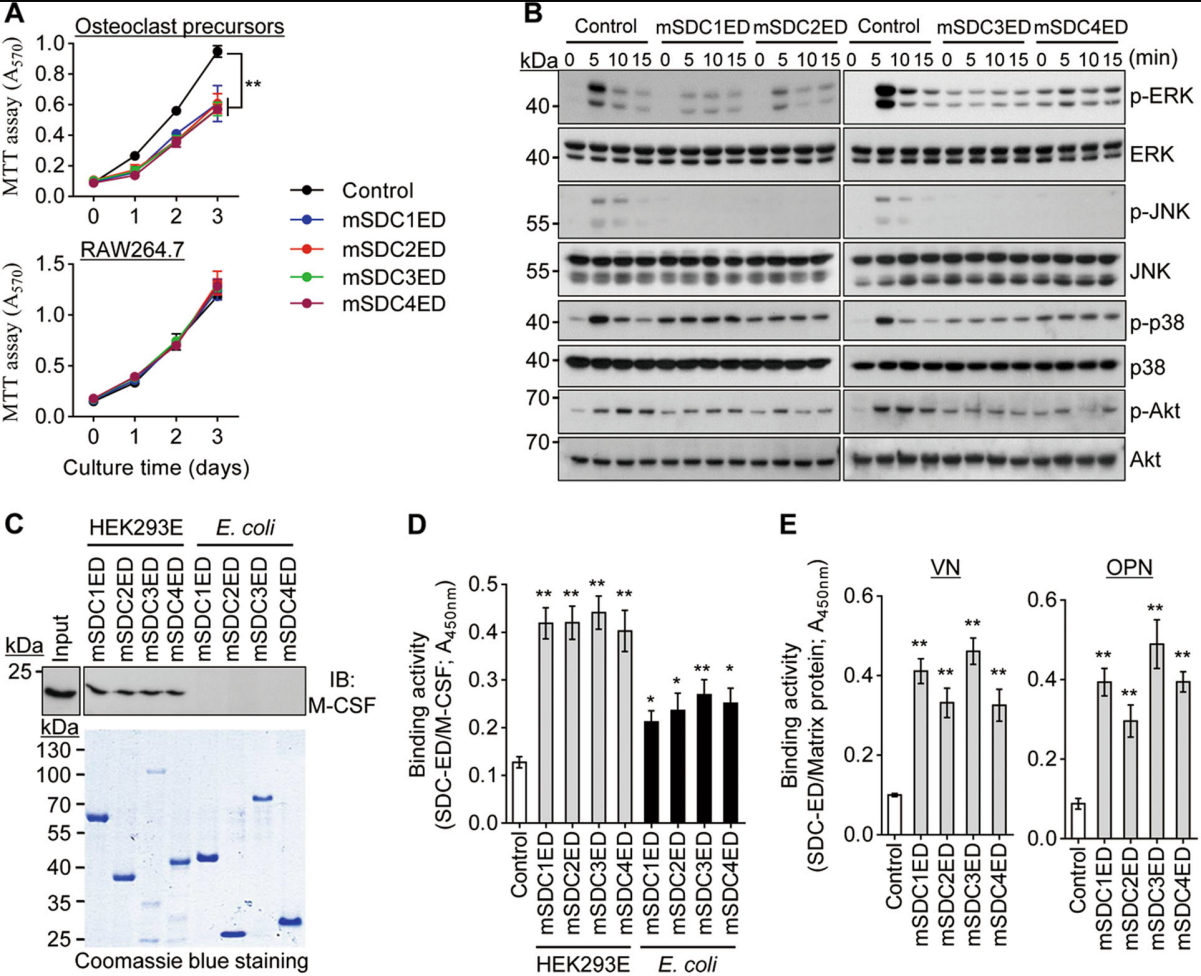
Syndecan ectodomains induce M-CSF malfunction. **a** Cell proliferation. Osteoclast precursors (upper panel) and RAW264.7 cells (lower panel) were treated with syndecan ectodomains (1 nM) in the presence and absence of M-CSF for 3 days, respectively. Cell proliferation was determined by MTT assay. **b** M-CSF-stimulated signaling. Osteoclast precursors were pre-incubated with syndecan ectodomains (10 nM) for 4 h and stimulated with M-CSF (5 ng/mL). Whole cell lysates were immunoblotted with specific antibodies against p-ERK, ERK, p-JNK, JNK, p-p38, p38, p-Akt, and Akt. **c** Pull-down assay. His-tagged syndecan ectodomains from HEK293E cells and *E. coli* immobilized on Ni-NTA agarose beads were incubated with recombinant M-CSF and centrifuged. Bound proteins were separated by 15% SDS-PAGE and analyzed with immunoblot analysis with anti-M-CSF antibody (upper panel). Syndecan ectodomains retained in the resultant pellets were resolved by 10% SDS-PAGE and detected by staining with Coomassie Blue (lower panel). **d** Immunosorbent-based assay. His-tagged syndecan ectodomains from HEK293E cells were attached to the Ni-coated plate and incubated with M-CSF. After washing, the binding affinity of M-CSF to syndecan ectodomains was quantitatively analyzed by sequential incubations with anti-M-CSF antibody, HRP-labeled secondary antibody, and substrate. Absorbance was measured at 450 nm wavelength. **e** Cross-linking between syndecan ectodomains and extracellular matrix proteins. His-tagged syndecan ectodomains from HEK293E cells were added to vitronectin (VN) and osteopontin (OPN)-coated plates and further incubated for 1 h. After washing, the bound proteins were quantitatively measured as described in (**d**) using anti-His antibody. Results represent the means ± SD (*n* *=* 4 as in **a**; *n* *=* 3 as in **d** and **e**). The *p* value indicates the comparison between the treatment group and control. *p < 0.05; **p < 0.01

Syndecans have been shown to be involved in the regulation of cell proliferation^16,24,26,27^. To analyze the inhibitory mechanisms of syndecan ectodomains on osteoclast differentiation, we first examined osteoclast precursor proliferation using MTT assay. Syndecan-1 to 4 ectodomains suppressed osteoclast precursor proliferation in the presence of M-CSF (Fig. 2a, upper panel). However, syndecan ectodomains had no effect on the growth of RAW264.7 cells, which are able to proliferate in the absence of M-CSF (Fig. 2a, lower panel), or on their differentiation in the presence of RANKL alone (Supplementary Fig. 3). In addition, syndecan-1 to 4 ectodomains strongly inhibited M-CSF-induced MAPKs (ERK, JNK, and p38) and Akt activation in osteoclast precursors (Fig. 2b), but did not affect RANKL-stimulated MAPKs activation (Supplementary Fig. 4). These findings suggest that the inhibitory effect of syndecan ectodomains on osteoclast differentiation (Fig. 1) may be related to the defect in M-CSF signaling.

The heparan sulfate chains of syndecan ectodomains are known to bind to growth factors, including HGF and FGF-2^16,17^. Thus, we investigated whether syndecan-1 to 4 ectodomains could interact with M-CSF using two independent binding assays. In the pull-down assay, the mixture of M-CSF and His-tagged syndecan ectodomains was bound to Ni-NTA agarose beads. M-CSF directly bound to syndecan ectodomains from HEK293E cells but not to those produced from bacteria (Fig. 2c). In another binding analysis, His-tagged syndecan ectodomains immobilized on a Ni-coated microtiter plate were reacted with M-CSF. The interaction of M-CSF with syndecan ectodomains from HEK293E cells was much stronger than its interaction with syndecan ectodomains from *E. coli* (Fig. 2d). These results indicate that syndecan ectodomains expressed in mammalian cells seem to directly bind to M-CSF via their heparan/chondroitin sulfate side chains. As sulfated GAG chains, including heparan/chondroitin sulfate, were reported to interact with extracellular matrix proteins^28–30^, we next examined whether syndecan ectodomains from HEK293E cells bind to matrix proteins such as vitronectin (VN) and osteopontin (OPN). Syndecan ectodomains effectively attached to VN and OPN upon their exposure to plates coated with VN or OPN (Fig. 2e). These results support the hypothesis that syndecan ectodomains bind to matrix proteins present in the bone marrow and that their complexes are able to recruit M-CSF to induce spatial sequestration of M-CSF in the extracellular compartment.

**Fig. 3:**
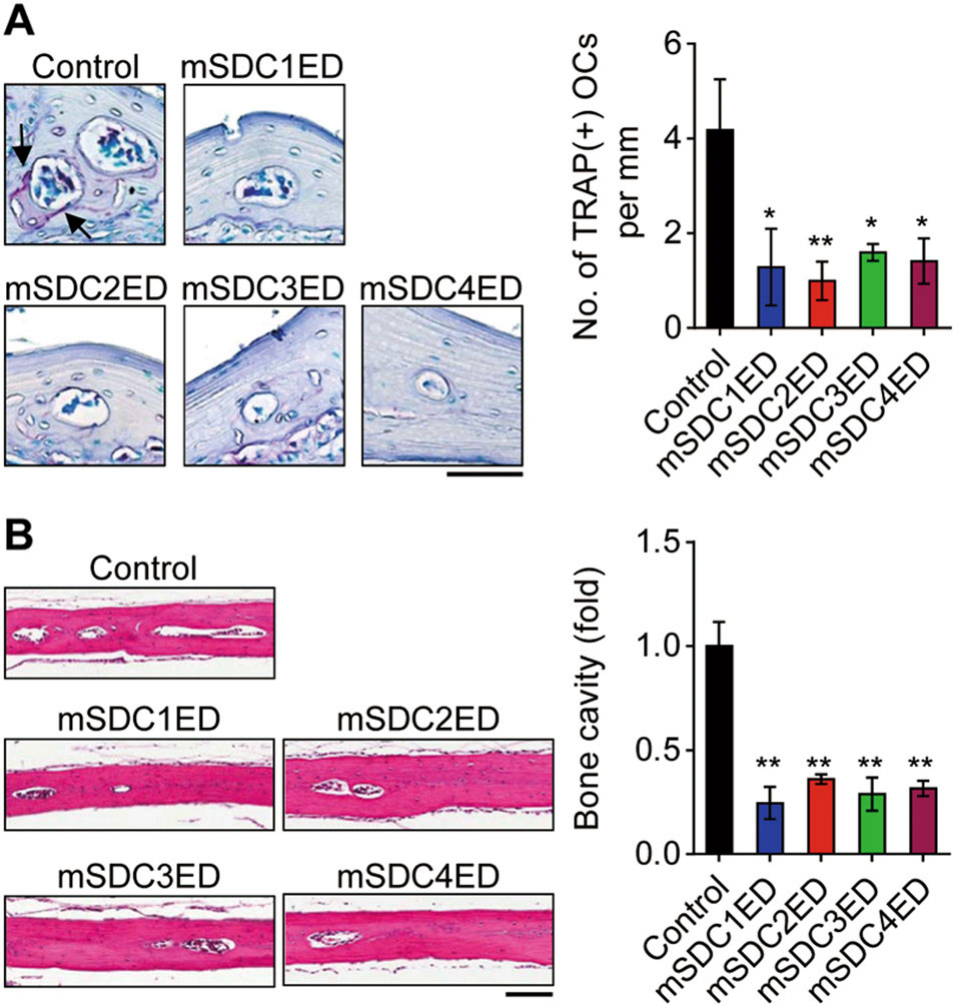
Syndecan ectodomains inhibit osteoclast formation and bone resorption *in vivo*. **a** Osteoclast formation in calvaria. Histological sections of the calvarial bone from mice injected with PBS (control) or syndecan-1 to 4 ectodomains (mSDC1ED to mSDC4ED) were stained for TRAP (left panel) and the number of TRAP-positive osteoclasts [TRAP(+) MNCs] was counted (right panel). Arrows indicate TRAP(+) OCs. **b** Bone cavity in calvaria. H&E-stained calvarial bone sections were photographed (left panel) and the area of bone cavity was quantified to evaluate osteoclastic bone resorption (right panel). Scale bar, 100 μm. Results represent the means ± SD (*n* *=* 4 as in **a**; *n* *=* 5 as in **b**). The *p* value indicates the comparison between the treatment group and control. *p < 0.05; **p < 0.01

To assess the effect of syndecan ectodomains on osteoclastogenesis in animal models, we introduced syndecan ectodomains from HEK293E cells into the periosteum of mouse calvaria. Consistent with in vitro data, syndecan-1 to 4 ectodomains decreased the formation of TRAP-positive osteoclasts on the surface of the calvarial bone (Fig. 3a). Furthermore, the area of calvarial bone marrow cavity, an index of bone resorption, was decreased following administration of syndecan ectodomains (Fig. 3b). These findings indicate that syndecan ectodomains inhibit osteoclast formation, thereby attenuating osteoclastic bone resorption in vivo.

### Heparan sulfate suppresses osteoclast differentiation via interaction with M-CSF

**Fig. 4:**
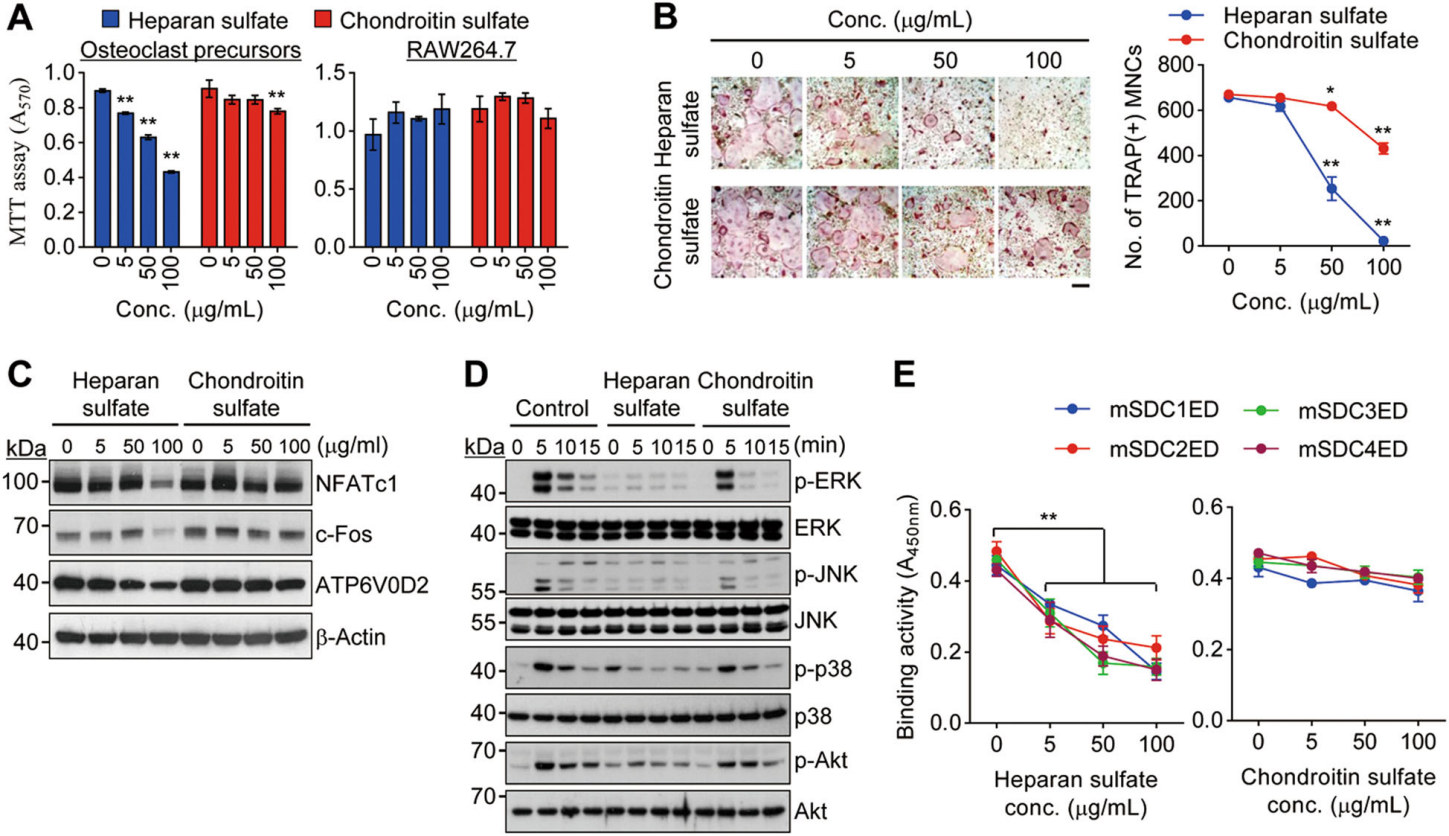
Heparan sulfate suppresses osteoclastogenesis through binding to M-CSF. **a** Cell proliferation. Osteoclast precursors (left panel) and RAW264.7 cells (right panel) were treated with heparan sulfate or chondroitin sulfate in the presence and absence of M-CSF for 3 days, respectively. Cell growth was measured by MTT assay. **b** Osteoclast differentiation. Osteoclast precursors were treated with either heparan sulfate or chondroitin sulfate and differentiated into osteoclasts for 4 days. Cells were stained for TRAP (left panel) and the number of TRAP(+) MNCs was counted (right panel). Scale bar, 200 μm. **c** Osteoclastogenic marker gene expression. Osteoclast precursors were treated with heparan sulfate or chondroitin sulfate and differentiated into osteoclasts for 3 days. The expression levels of the osteoclast markers (NFATc1, c-Fos, and ATP6V0D2) were assessed by immunoblot analysis. β-Actin was used as a loading control. **d** M-CSF-dependent signaling. Osteoclast precursors were incubated with heparan sulfate or chondroitin sulfate (100 μg/mL) for 4 h and then stimulated with M-CSF (5 ng/mL). The activity of M-CSF-dependent signaling was examined by immunoblot analysis. **e** Competitive binding assay. A pre-mixture of M-CSF and heparan sulfate or chondroitin sulfate at various concentrations was incubated with His-tagged syndecan ectodomains from HEK293E cells bound on the Ni-coated plate. After washing, the level of M-CSF on Ni-coated plate was analyzed as described in Fig. 2d to assess the binding affinity of M-CSF to syndecan ectodomains. Results represent the means ± SD (*n* *=* 4 as in **a**; *n* *=* 3 as in **b** and **e**). The *p* value indicates the comparison between the treatment group and control. *p < 0.05; **p < 0.01

Previous reports have shown that syndecan ectodomains contain heparan sulfate and chondroitin sulfate chains that regulate cell growth and differentiation^16,26^. We first analyzed osteoclast proliferation in two different cell systems, bone marrow-derived osteoclast precursors and RAW264.7 cells growing in the presence and absence of M-CSF, respectively. Heparan sulfate effectively inhibited osteoclast precursor proliferation as compared with chondroitin sulfate (Fig. 4a, left panel). However, both heparan sulfate and chondroitin sulfate had no effect on the growth of RAW264.7 cells (Fig. 4a, right panel). Heparan sulfate strongly diminished the differentiation of osteoclast precursors into osteoclasts in a dose-dependent manner, but chondroitin sulfate had no remarkable effect on the inhibition of osteoclast differentiation (Fig. 4b). This phenomenon was further confirmed by the analysis of the expression of osteoclastogenic genes, including NFATc1, c-Fos, and ATP6V0D2 (Fig. 4c). Furthermore, M-CSF-induced phosphorylation of MAPKs (ERK, JNK, and p38) and Akt was blocked by heparan sulfate but not chondroitin sulfate (Fig. 4d). These findings suggest that the inhibition of osteoclast precursor proliferation and differentiation by heparan sulfate may be associated with M-CSF malfunction. To determine the causative factor responsible for the defect in M-CSF function, we performed a competition binding assay. The binding of syndecan-1 to 4 ectodomains to M-CSF was significantly inhibited by heparan sulfate, but not by chondroitin sulfate, in a concentration-dependent manner (Fig. 4e). Also, the binding of syndecan ectodomains to VN and OPN was inhibited by heparan sulfate, suggesting an interaction between heparan sulfate and extracellular matrix proteins (Supplementary Fig. 5). Overall, these results indicate that heparan sulfate chains attached to syndecan ectodomains are associated with the negative regulation of osteoclastogenesis through direct binding to M-CSF and extracellular matrix proteins.

### 2-O and 6-O-sulfate groups of heparin are critical for the inhibition of osteoclast differentiation

**Fig. 5:**
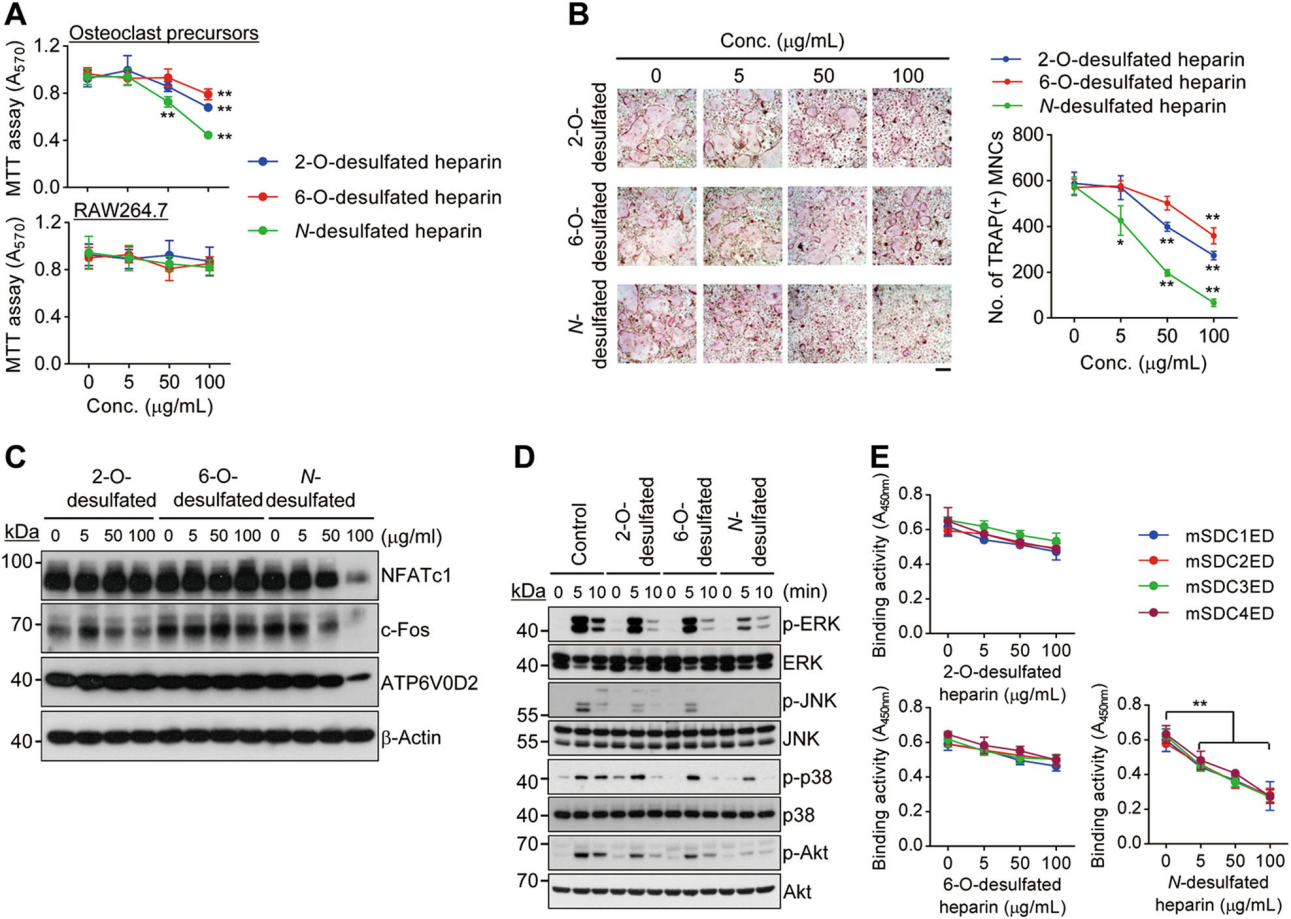
*N*-desulfated heparin with 2-O and 6-O-sulfated groups effectively binds to M-CSF and inhibits osteoclast differentiation. **a** Cell proliferation. Osteoclast precursors (upper panel) and RAW264.7 cells (lower panel) were treated with 2-O, 6-O-, or *N*-desulfated heparin in the presence and absence of M-CSF for 3 days, respectively. Cell proliferation was determined by MTT assay. **b** Osteoclast differentiation. Osteoclast precursors were treated with 2-O, 6-O-, or *N*-desulfated heparin and differentiated into osteoclasts. TRAP-stained cells were photographed (right panel) and TRAP(+) MNCs were counted (left panel). Scale bar, 200 μm. **c** Gene expression of osteoclastogenic markers. Osteoclast precursors were treated with 2-O, 6-O-, or *N*-desulfated heparin and differentiated into osteoclasts for 3 days. The expression levels of osteoclast markers were examined by immunoblot analysis. β-Actin was used as a loading control. **d** M-CSF-specific signaling. M-CSF-induced signals were analyzed as described in Fig. 4d using 2-O, 6-O-, or *N*-desulfated heparin. **e** Quantitative binding affinity of M-CSF to syndecan ectodomains was analyzed with a competition assay using 2-O, 6-O-, or *N*-desulfated heparin as described in Fig. 4e. Results represent the means ± SD (*n* *=* 4 as in **a**; *n* *=* 3 as in **b** and **e**). The *p* value indicates the comparison between the treatment group and control. *p < 0.05; **p < 0.01

Despite the inhibitory action of heparan sulfate in osteoclastogenesis, its clinical use in bone repair may be restricted owing to its anticoagulant activity. We observed that heparan sulfate exhibited anticoagulant activity in a dose-dependent manner (Supplementary Fig. 6). Heparin was reported to be chemically related to heparan sulfate, displaying the same disaccharide backbone^11^. It is well established that both heparan sulfate and heparin could equally activate FGF signaling by binding to FGFs and their receptors^31,32^. Moreover, desulfated heparin derivatives showed no detectable anticoagulant activity as compared with heparin^33,34^. Based on these facts, we investigated the effects of various desulfated heparin derivatives on osteoclast precursor proliferation and differentiation. *N*-desulfated heparin with no anticoagulant activity (Supplementary Fig. 6) showed higher inhibition of M-CSF-dependent osteoclast precursor proliferation than 2-O or 6-O-desulfated heparin (Fig. 5a, upper panel); we failed to observe this phenomenon in M-CSF-independent RAW264.7 cells (Fig. 5a, lower panel). In addition, *N*-desulfated heparin markedly suppressed osteoclast differentiation as compared to 2-O and 6-O-desulfated heparin (Fig. 5b), with a concurrent decrease in the expression of osteoclastogenic genes as well as M-CSF-induced osteoclastogenic signaling (Figs. 5c, d). To test the interaction between various desulfated forms of heparin and M-CSF, His-tagged syndecan ectodomains were immobilized on a Ni-coated plate and incubated with M-CSF and various forms of desulfated heparin. *N*-desulfated heparin strongly restrained the binding between syndecan ectodomains and M-CSF as compared to 2-O- or 6-O-desulfated heparin (Fig. 5e). Also, *N*-desulfated heparin inhibited binding of syndecan ectodomains to VN and OPN, supporting the notion that *N*-desulfated heparin interacts with extracellular matrix proteins (Supplementary Fig. 7). Our findings demonstrate that *N*-desulfated heparin with 2-O and 6-O-sulfated groups and no anticoagulant activity in blood may inhibit osteoclastogenesis by binding to M-CSF and extracellular matrix proteins.

### Heparan sulfate and *N*-desulfated heparin inhibit osteoclast formation and bone resorption in vivo

**Fig. 6:**
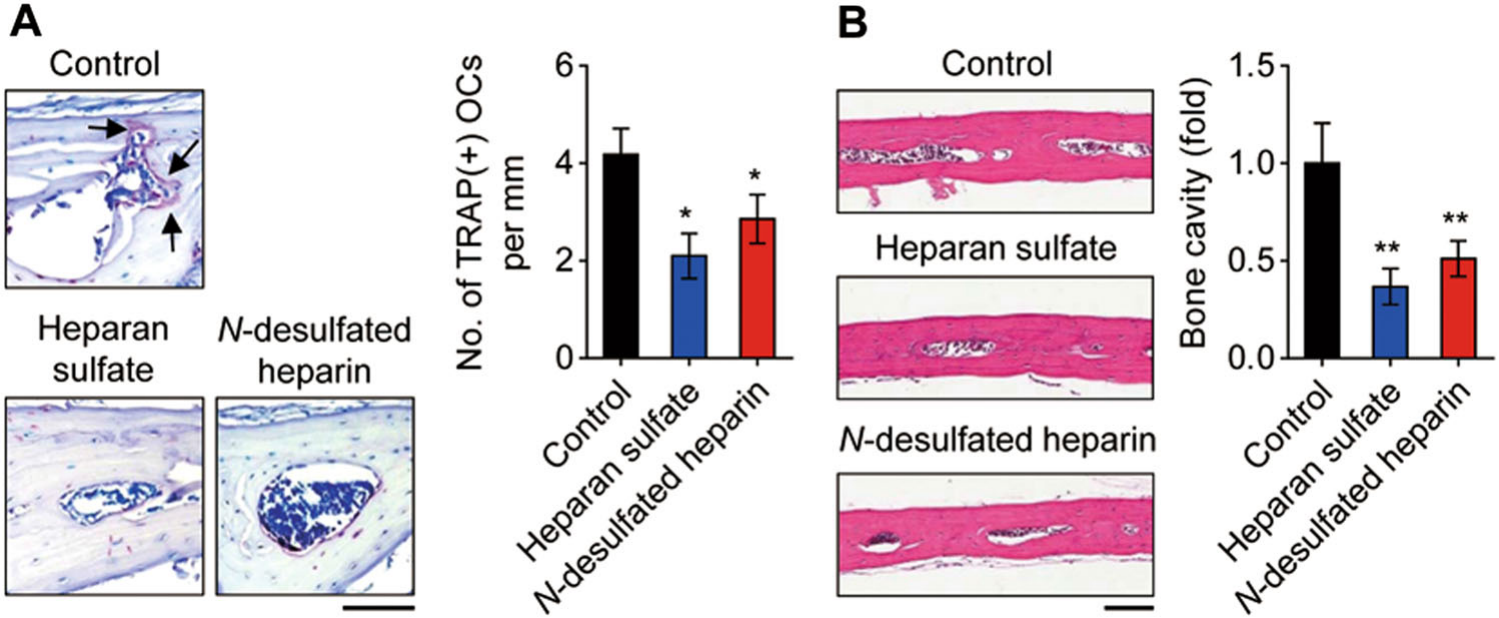
Heparan sulfate and *N*-desulfated heparin suppress osteoclast formation in vivo. **a** Osteoclast formation. Histological sections of calvarial bone from mice injected with PBS (control), heparan sulfate, or *N*-desulfated heparin were stained with TRAP and the number of TRAP-positive osteoclasts [TRAP(+) OCs] was counted on the surface of the calvarial bone. Scale bar, 100 μm. **b** Calvarial bone marrow cavity. The extent of bone marrow cavity in H&E-stained calvarial bone sections was quantitatively analyzed to assess osteoclast bone resorption activity. Scale bar, 100 μm. Results represent the means ± SD (*n* *=* 4 as in **a**; *n* *=* 5 as in **b**). The *p* value indicates the comparison between the treatment group and control. *p < 0.05; **p < 0.01

As shown in Fig. 4 and Fig. 5, we observed that heparan sulfate and *N*-desulfated heparin inhibited osteoclast differentiation in vitro. We, therefore, analyzed the effect of heparan sulfate and *N*-desulfated heparin on osteoclast formation in vivo. The administration of heparan sulfate and *N*-desulfated heparin to calvarial periosteum of mice daily for 5 days resulted in a decrease in TRAP-positive mature osteoclast formation on the surface of the calvarial bone by ~50 and ~32% relative to control, respectively (Fig. 6a). In addition, the histological analysis of calvarial bone sections stained with H&E revealed that the area of calvarial bone marrow cavity, which represents osteoclastic bone resorption activity, was significantly decreased in heparan sulfate and *N*-desulfated heparin-challenged mice as compared to control mice (Fig. 6b). Consistent with this, the bone-resorptive activity of mature osteoclasts in vitro was significantly reduced by treatment with ectodomains of syndecan-1, 2, and 4 from HEK293E cells, heparan sulfate, and *N*-desulfated heparin compared to the controls (Supplementary Fig. 8). Thus, heparan sulfate and *N*-desulfated heparin negatively regulated osteoclast differentiation in vivo, thereby suppressing osteoclastic bone resorption.

## Discussion

This is the first study to demonstrate that syndecan ectodomains originated from mammalian cells, heparan sulfate chains, and *N*-desulfated heparin derivatives could directly interact with M-CSF and decrease osteoclast formation in vitro and in vivo. This inhibitory effect was attributed to the functional blocking of M-CSF-mediated osteoclastogenic signaling. Syndecan ectodomains, heparan sulfate chains, and *N*-desulfated heparin are thought to play a role as a soluble decoy receptor for M-CSF and may compete with M-CSF receptor, c-Fms, for binding to M-CSF in the extracellular compartment. Some decoying factors for M-CSF may disturb the osteoclastogenic signaling via M-CSF-mediated c-Fms axis. We also found that syndecan ectodomains directly bind to extracellular matrix proteins. Thus, the combined results show that the shed ectodomains of syndecans bound to extracellular matrix proteins recruit M-CSF via heparan/chondroitin sulfate side chains and the defective M-CF signaling caused by the decreased binding opportunity between M-CSF and c-Fms retards osteoclast differentiation.

Syndecan-1 ectodomains derived from tumor cells have been reported to exhibit opposite effects on osteoclastogenesis. Syndecan-1 ectodomain purified from multiple myeloma cells significantly inhibited osteoclast differentiation at picomolar concentrations^26^. In contrast, syndecan-1 ectodomains in conditioned medium from breast cancer cells played a critical role in the stimulation of osteoclastogenesis in human peripheral blood mononuclear cells^18^. Our findings showed that murine ectodomains of syndecan-1 to 4 expressed from human embryonic kidney cells inhibited the differentiation of murine bone marrow-derived osteoclast precursors. We and others have demonstrated that heparan sulfate chains of syndecan ectodomains are essential for osteoclastogenesis regulation, but these results were controversial as described above. Heparan sulfate chains of syndecan ectodomains have been found to exhibit molecular polymorphism owing to different post-translational modifications. Thus, heparan sulfate chains with distinct functions could be produced in a specific cell or tissue type during different physiological states, neoplastic transformation, and lung development^19,35–37^. Tumor heparan sulfate chains were reported to differ in their sizes, fine structures, and ligand affinities from heparan sulfate chains produced in the corresponding normal tissue^9^. Thus, the controversial effects of syndecan ectodomains on osteoclastogenesis may be explained through the structural polymorphism of their heparan sulfate chains.

It has been reported that the ectodomain of the mouse syndecan-1 core protein produced in bacteria inhibited the spreading and migration of human breast carcinoma cells through the formation of a signaling complex with integrin αvβ3^38^. Syndecan-1 core protein was reported to contain extracellular regulatory sites required for its interaction with integrin αvβ3^39^. In osteoclasts, integrin αvβ3 plays a critical role in controlling cell adhesion, migration, and differentiation^40,41^, suggesting that bacterially expressed core proteins of syndecan ectodomains may participate in the regulation of osteoclast differentiation through the interaction with, and activation of, integrin αvβ3. In addition, we observed that bacterially produced syndecan ectodomains lacking heparan sulfate chains inhibited osteoclast differentiation. This inhibitory effect of core proteins of syndecan ectodomains is thought to regulate osteoclastogenesis regardless of M-CSF.

Heparin, one of the most important anticoagulant drugs in the clinic for a long time, inhibits tumor development and metastasis and limits several inflammatory diseases^11,42,43^. The clinical use of heparin in the development of drugs for inflammatory diseases and cancers may be limited by its anticoagulant activity^43^. Thus, the anticoagulant activity should be considered for the development of heparin as a drug. Selectively desulfated forms of heparin were reported to retain the anti-inflammatory and anti-tumor functions with minimal anticoagulant effects^33,34,44^. In this study, we revealed that selectively *N*-desulfated heparin with no detectable anticoagulant activity may be useful for anti-osteoporotic therapy, reducing the risk of unwanted hemorrhagic side effects.

Sulfation was known to play a critical role in the biological activities of heparin and heparan sulfate and regulates their binding to various proteins and growth factors through electrostatic interaction^45,46^. In heparin and heparan sulfate, sulfation mainly occurs at N, 2-O, and 6-O-positions^47,48^ and growth factors recognize specific sulfate groups^49^. For instance, 2-O-sulfate groups of heparin were essential for the binding to FGF-2, and 6-O-sulfate groups mediate binding to the receptor, FGFR-1^50^. The 6-O-sulfate groups of heparan sulfate are necessary for the interaction with HGF^51^ and the binding affinity of 2-O-desulfated heparin for bone morphogenetic protein-2 was greater than that of heparin^52^. In line with the fact highlighting the distinct role of specific sulfate groups of heparin/heparan sulfate in binding to cytokines or growth factors, our study showed that *N*-desulfated heparin with 2-O- and 6-O-sulfate groups interacts with M-CSF and consequently suppress osteoclast differentiation. Osteoprotegerin (OPG), a soluble decoy receptor for RANKL, was shown to bind to heparin and heparan sulfates^53^. Heparin was reported to indirectly enhance osteoclast differentiation via binding to OPG secreted by osteoblasts^54^. Intact heparin and 2-O-desulfated heparin may bind to OPG and stimulate osteoclastogenesis, but *N*-desulfated heparin showed no interaction with OPG and failed to activate osteoclastogenesis, indicative of the important role of *N*-sulfate groups of heparin in osteoclast differentiation^54^. Considering the broad range of heparin-binding proteins, it is not surprising that intact heparin may interact with both M-CSF and OPG to exert opposite effects on osteoclastogenesis. As the anticoagulant activity of intact heparin limits its clinical use, selectively desulfated heparin derivatives with no anticoagulant effects may offer advantages for targeting metabolic bone diseases. Based on our results, *N*-desulfated heparin that suppresses osteoclast differentiation and osteoclastic bone resorption (via binding to M-CSF) may be used as a therapeutic agent for osteoporotic bone loss. On the other hand, 2-O-desulfated heparin that stimulates osteoclastogenesis via binding to OPG may be useful for the treatment for osteopetrosis or serve as a potential drug to halt the progression of osteopetrosis. As heparin and heparan sulfate were reported to inhibit osteoclastogenesis in RAW 264.7 cells in the absence of M-CSF and OPG^55^, we suggest that heparin and heparan sulfate chains may interact with other factors involved in the regulation of osteoclast differentiation.

In conclusion, we showed that syndecan ectodomains, heparan sulfate, and *N*-desulfated heparin exert suppressive effects on osteoclastogenesis and osteoclastic bone resorption. In particular, selectively *N*-desulfated heparin derivatives may be clinically useful as anti-resorptive agents for osteoporosis without side effects associated with the anticoagulant action.

## Materials and methods

### Cell culture and antibodies

Human embryonic kidney 293E (HEK293E) cells were maintained in Dulbecco’s modified Eagle’s medium (DMEM, Invitrogen, Carlsbad, CA, USA) supplemented with antibiotics and 5% fetal bovine serum (FBS, Invitrogen) in the presence of M-CSF. RAW264.7 cell, a murine macrophage cell line, was cultured in DMEM supplemented with antibiotics and 10% FBS in the absence of M-CSF. Antibodies used in this study were as follows: His antibody from Roche; NFATc1, c-Fos, ATP6V0D2, actin, p-Akt, and Akt antibodies from Santa Cruz Biotechnology (Santa Cruz, CA, USA); M-CSF antibody from Abcam; and p-ERK, ERK, p-JNK, JNK, p-p38, and p38 antibodies from Cell Signaling Technology (Boston, MA, USA).

### Construction, expression, and purification of syndecan ectodomains

For bacterial expression of mouse syndecan ectodomains, the cDNAs for syndecan ectodomains were obtained by polymerase chain reaction (PCR) using primers listed in Supplementary Table 1. The PCR products were digested with *Nhe*I/*Not*I and inserted into the corresponding sites of pET28a vector. *Escherichia coli* Rosetta 2 (DE3) cells (Novagen, Darmstadt, Germany) were transformed with the pET28a-syndecan ectodomain constructs. The expression of syndecan ectodomains was induced with the addition of 0.25 mM isopropyl-β-D-thiogalactopyranoside (BioBasic Inc., Amherst, NY, USA) at 25 °C for 18 h. Harvested cells were lysed in a lysis buffer (20 mM Tris-HCl, pH 7.5, and 1 mg/mL lysozyme) by sonication and the cell lysates were bound to Ni-NTA agarose beads (ELPIS Biotech). The beads were washed twice with a washing buffer [20 mM Tris-HCl, pH 7.5, 0.5 M sodium chloride (NaCl), and 60 mM imidazole] and eluted with an elution buffer (20 mM Tris-HCl, pH 7.5, 0.5 M NaCl, and 0.5 M imidazole). The eluted proteins were desalted and exchanged with phosphate-buffered saline (PBS) using ultrafiltration system (10-kDa cut-off; PALL Life Sciences, Port Washington, NY, USA). For the mammalian expression of mouse syndecan ectodomains, the cDNAs of syndecans were amplified by PCR using primers listed in Supplementary Table 2. The PCR products were digested with *Eco*RI/*Not*I and ligated into the correct reading frames of the mammalian expression vector pIRES. HEK293E cells were transfected with pIRES-syndecan ectodomain vectors and cultured with DMEM supplemented with 1% FBS and 2 μg/mL puromycin (AMRESCO, Solon, OH, USA). The culture medium was collected every other day for 2 weeks. The collected media were concentrated and exchanged with a buffer containing 20 mM Tris-HCl, pH 7.5, 0.5 M NaCl, and 10 mM imidazole using ultrafiltration. After incubation with Ni-NTA agarose beads, the beads were washed and eluted with an elution buffer (20 mM Tris-HCl, pH 7.5, 0.5 M NaCl, and 0.5 M imidazole). The purified recombinant proteins were concentrated and dialyzed with PBS using ultrafiltration.

### Osteoclast differentiation and bone pit formation

Bone marrow-derived cells were collected from the femurs and tibias of 6-week-old C57BL6 male mice (Central Lab Animal; Seoul, Korea) by flushing the bone marrow. Cells were treated with a red blood cell lysis buffer (Sigma-Aldrich, St. Louis, MO, USA). The remaining cells were incubated with α-MEM supplemented with 10% FBS and M-CSF (5 ng/mL) for 12 h. Floating cells were harvested and cultured with M-CSF (30 ng/mL) for 3 days to generate osteoclast precursors. Osteoclast differentiation was induced by culturing osteoclast precursors with M-CSF (30 ng/mL) and RANKL (100 ng/mL) for 4 days. Additionally, RAW264.7 cells were induced to differentiate into osteoclasts by treatment with RANKL (100 ng/mL) for 4 days. TRAP staining was performed using a leukocyte acid phosphatase staining kit (Sigma-Aldrich) as per the manufacturer’s protocol and TRAP-positive multinucleated cells [TRAP(+) MNCs] with more than three nuclei were counted using a light microscope. For TRAP activity assay, osteoclast precursors (4 × 10^4^ cells/well) were seeded into a 24-well culture plate and cultured with M-CSF and RANKL for 2 days. The cells were lysed with TRAP buffer (120 mM sodium acetate, pH 5.2, and 12 mg/mL sodium tartrate) containing 1% Triton X-100. After centrifugation, the supernatant was mixed with *p*-nitrophenyl phosphate solution (Sigma-Aldrich) and incubated at 37 °C for 30 min. The reaction was stopped by adding 1 N sodium hydroxide (NaOH) and the absorbance was measured at 405 nm wavelength using a microplate reader (Bio-Rad, Hercules, CA, USA). For the bone resorption assay, mature osteoclasts (0.5 × 10^3^ cells/well in a 96-well culture plate) were detached from the culture dish using a cell dissociation solution (Sigma-Aldrich), seeded onto dentine slices (Immunodiagnostic Systems Ltd., Boldon, UK) and further incubated with α-MEM containing M-CSF (30 ng/mL) and RANKL (100 ng/mL) for 2 days to allow bone resorption. Cells were then removed from the dentine slices by ultrasonication, and the slices were stained with hematoxylin (Sigma-Aldrich). The area of resorbed pits was analyzed using a light microscope and Image-Pro Plus version 6.0 software (MediaCybermetics, Silver Spring, MD, USA).

### Evaluation of cell proliferation with 3-(4,5-dimethylthiazol-2-yl)-2,5-diphenyltetrazolium bromide (MTT) assay

For cell proliferation assay, bone marrow-derived osteoclast precursors and RAW264.7 cells (0.5 × 10^4^ cells/well) were plated on a 48-well plate and treated with syndecan ectodomains, heparan sulfate, and desulfated heparin derivatives for indicated times. Cells were treated with MTT solution (0.5 mg/mL in PBS; Sigma-Aldrich) at 37 °C for 1 h. Formazan crystals were dissolved in dimethyl sulfoxide and the absorbance was measured at 570 nm using a microplate reader (Bio-Rad).

### Measurement of anticoagulant activity

The anticoagulant activity of heparan sulfate or heparin derivatives was measured using the Anti-Xa heparin test kit (Iduron, Cheshire, UK) according to the manufacturer’s protocol. Heparan sulfate (Sigma-Aldrich) or desulfated heparin derivatives (Iduron) were incubated with anti-thrombin solution (0.5 IU/mL) for 2 min at 37 °C. Cells were treated with Factor Xa (2.5 μg/mL) for 2 min at 37 °C. Factor Xa substrate (0.625 mg/mL) was added to develop color and the reaction was stopped with 20% acetic acid. The absorbance was measured at 405 nm using a microplate reader (Bio-Rad).

### In vitro binding assay

For the analysis of the interaction between syndecan ectodomains and M-CSF using a pull-down method, recombinant human M-CSF (1 μg, 146 amino acid residues between amino acids 36 and 181) was incubated with His-syndecan ectodomains (1 μg) immobilized on Ni-NTA agarose beads (ELPIS-Biotech) in a binding buffer [20 mM Tris-HCl, pH 7.3, 150 mM potassium chloride (KCl), 0.2 mM ethylenediaminetetraacetic acid (EDTA), 1 mM dithiothreitol, 20% glycerol, 0.1% Nonidet P-40, and 1 mM phenylmethylsulfonyl fluoride] at 4 °C for 12 h. The beads were washed five times using a binding buffer and centrifuged. M-CSF present in the pellet was detected by immunoblot analysis with a specific antibody against M-CSF.

For the analysis of the interaction between syndecan ectodomains and M-CSF using an immunosorbent-based assay, His-tagged syndecan ectodomains (10 pmole/well) were incubated with Ni-coated 96-well plates (Pierce, Rockford, IL, USA) at 4 °C for 12 h. The plates were washed with a washing buffer (20 mM Tris-HCl, pH 7.3, 150 mM NaCl, and 0.05% Tween-20) and blocked with a binding buffer [20 mM Tris-HCl, pH 7.3, 150 mM NaCl, 0.1% bovine serum albumin (BSA), and 0.05% Tween-20] for 1 h. The plates were further incubated with recombinant M-CSF (20 pmol/well) and washed with the binding buffer, followed by treatment with horseradish peroxidase (HRP)-labeled secondary antibody and 3,3′,5,5′-tetramethylbenzidine (TMB) substrate (Pierce). The binding affinity of M-CSF to syndecan ectodomains was analyzed by measuring the absorbance at 450 nm wavelength.

For the evaluation of the interaction between syndecan ectodomains and extracellular matrix proteins, 96-well culture plates were coated with VN (10 μg/mL, BD Biosciences, San Jose, CA, USA) or OPN (10 μg/mL, Sigma-Aldrich) at 4 °C for 12 h. The coated plates were blocked with the binding buffer (20 mM Tris-HCl, pH 7.3, 150 mM NaCl, 0.1% BSA, and 0.05% Tween-20) for 1 h at room temperature and washed with the washing buffer. The plates were incubated with His-tagged syndecan ectodomains from HEK293E cells (10 pmol/well) for 2 h at room temperature and sequentially exposed to anti-His antibody, HRP-labeled secondary antibody, and substrate. The binding affinity was analyzed by measuring the absorbance at 450 nm wavelength.

For the competition binding assay, a mixture of M-CSF (20 pmol/well) and heparan sulfate, chondroitin sulfate (Sigma-Aldrich), or desulfated heparin derivatives was incubated with His-tagged syndecan ectodomains (10 pmol/well) bound onto Ni-coated 96-well plates for 2 h at room temperature. To detect M-CSF bound to His-tagged syndecan ectodomains immobilized on Ni-coated plates, the plates were washed and incubated with anti-M-CSF antibody and HRP-labeled anti-rabbit IgG. The plates were washed five times and developed with TMB substrate (Pierce). The reaction was stopped by adding 5 N sulfuric acid (H_2_SO_4_) and the absorbance was read at 450 nm using a microplate reader (Bio-Rad).

### Animal studies

C57BL6 male mice were purchased from Central Lab Animal (Seoul, Korea) and maintained at the animal facility of Yeungnam University College of Medicine. All animal experiments were approved by the institutional review board of Yeungnam University Medical Center and were in compliance with the Guide for the Care and Use of Laboratory Animals. Mice at 8 weeks of age were subcutaneously injected with ectodomains of recombinant mouse syndecan-1 to 4 (0.2 mg/kg in 100 μL PBS), heparan sulfate (10 mg/kg in 100 μL PBS), heparin derivatives (10 mg/kg in 100 μL PBS), or PBS alone (control) over the calvaria every day for 5 days. After 4 days, mice were euthanized and calvaria were collected for the evaluation of osteoclast formation and bone cavity. The specimens fixed with formaldehyde were embedded in paraffin and sectioned. Paraffin-embedded sections of calvaria were deparaffinized and stained with TRAP to analyze osteoclast formation, and H&E staining was performed to assess bone marrow cavity. To measure bone cavity, images were scanned with an Aperio ScanScope Model T3 and analyzed with ImageScope software (Aperio Technologies, Vista, CA, USA).

### Statistical analysis

All data are presented as mean ± standard deviation (SD) from at least three independent experiments. Data with more than three groups were evaluated using analysis of variance, followed by Bonferroni’s comparison test between two groups with GraphPad Prism (GraphPad Software Inc., La Jolla, CA, USA). For all experiments, *p* < 0.05 indicated significance.

## Electronic supplementary material

Supplementary Figures

Supplementary Tables
