# Supplementary material for: Suppressive effect of syndecan ectodomains and N-desulfated heparins on osteoclastogenesis via direct binding to macrophage-colony stimulating factor
Source: Cell Death Dis. 2018 Nov 2;9(11):1119. doi: 10.1038/s41419-018-1167-8 (PMC6215006; doi:10.1038/s41419-018-1167-8)
Supplement: Supplementary file 1 — Supplementary Figures [file 41419_2018_1167_MOESM1_ESM.docx]

**SUPPLEMENTARY INFORMATION**

**Supplementary Figures**


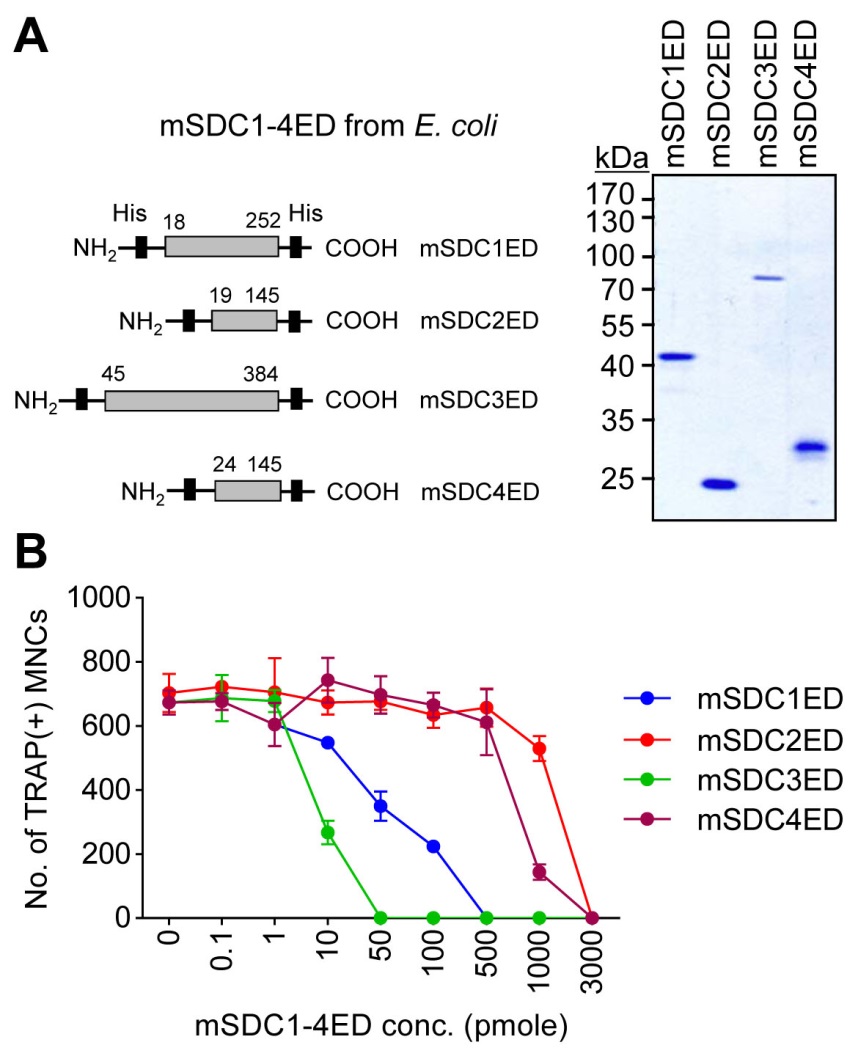


**Supplementary Fig. 1 Syndecan ectodomains produced from *E. coli* inhibit osteoclast differentiation. a** Schematic diagram of mouse syndecan-1 to -4 ectodomains (mSDC1ED to mSDC4ED) expressed in *E. coli*. The gray and black boxes indicate the syndecan ectodomain and histidine (His), respectively (left panel). Recombinant mouse syndecan-1 to -4 ectodomains purified from *E. coli* were assessed by 10% SDS-PAGE and stained with Coomassie Brilliant Blue (right panel). **b** Osteoclast precursors were treated with the indicated amount of recombinant syndecan-1 to -4 ectodomains from *E. coli* and differentiated into osteoclasts for 4 days. Cells were stained for TRAP and the number of TRAP(+) MNCs was counted.

**
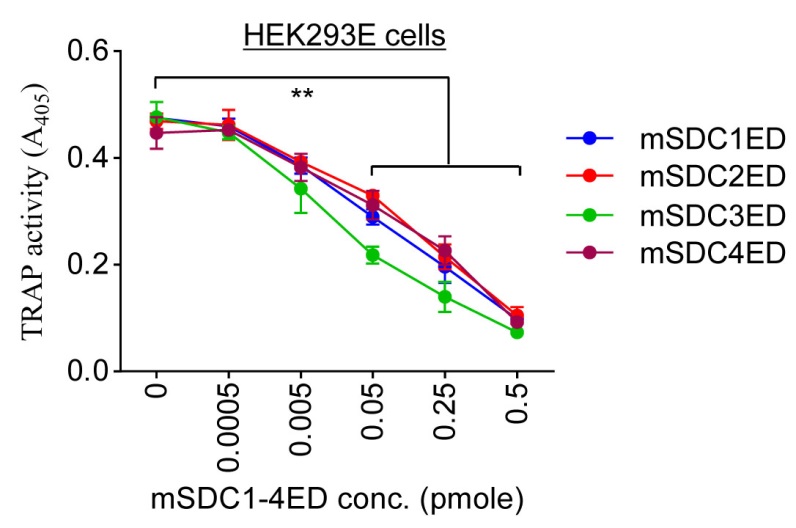
**

**Supplementary Fig. 2 Change in TRAP activity by syndecan ectodomains.** Osteoclast precursors were treated with syndecan ectodomains from HEK293E cells and further differentiated into osteoclasts in the presence of M-CSF (30 ng/mL) and RANKL (100 ng/mL) for 48 h. To assess the efficacy of syndecan ectodomains in the early stage of osteoclast differentiation, the activity of TRAP was measured by reading the absorbance at 405 nm wavelength. Results represent the means ± SD (*n* = 3). ***p* < 0.01.

**
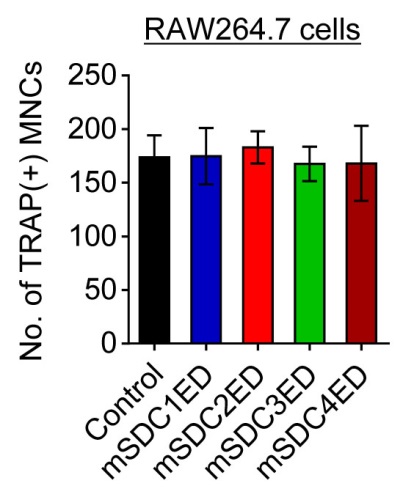
**

**Supplementary Fig. 3 Effect of syndecan ectodomains on differentiation of RAW264.7 cells into osteoclasts.** RAW264.7 cells were treated with syndecan-1 to -4 ectodomains (mSDC1ED to mSDC4ED, 1 nM) and differentiated into osteoclasts in the presence RANKL (100 ng/mL) for 4 days. Cells were stained for TRAP and the number of TRAP(+) MNCs was counted.

**
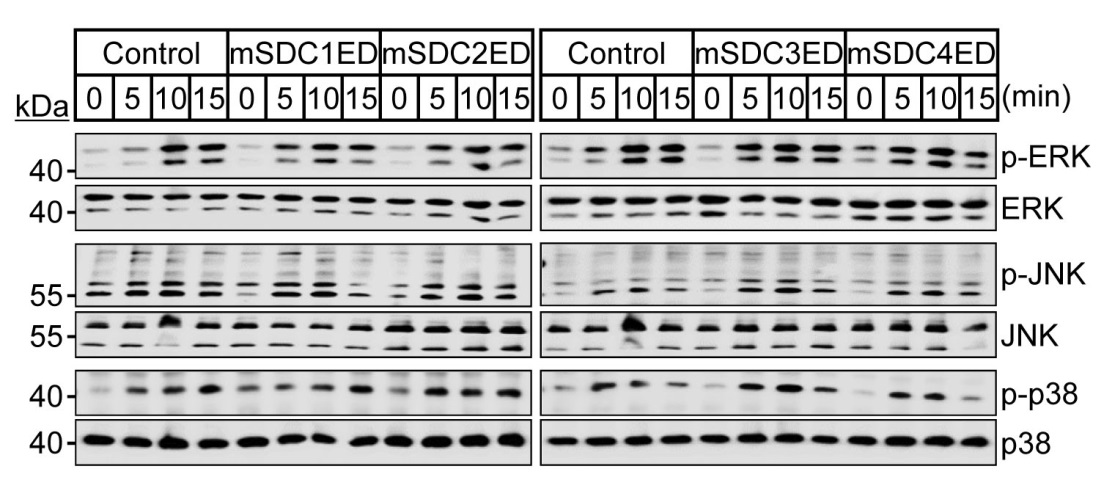
**

**Supplementary Fig. 4 Effect of syndecan ectodomains on RANKL-stimulated MAPKs signaling.** Osteoclast precursors were pre-incubated with syndecan ectodomains (10 nM) for 4 h and stimulated with RANKL (100 ng/mL). Whole extracts were used for immunoblotting with specific antibodies against p-ERK, ERK, p-JNK, JNK, p-p38, and p38.

**
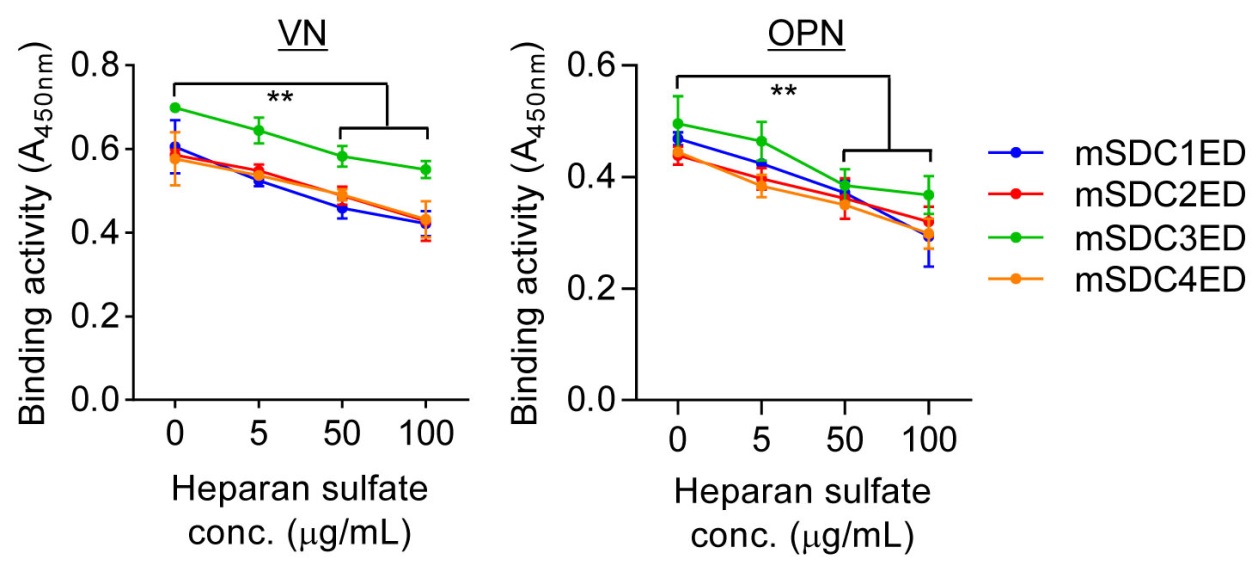
**

**Supplementary Fig. 5 Binding of heparan sulfate to vitronectin (VN) or osteopontin (OPN).** To assess the interaction between heparan sulfate and extracellular matrix proteins, 96-well culture plates were coated with VN (10 μg/mL) or OPN (10 μg/mL) at 4°C for 12 h and washed with wash buffer (20 mM Tris-HCl, pH 7.3, 150 mM NaCl, and 0.05% Tween-20). Coated plates were blocked with binding buffer [20 mM Tris-HCl, pH 7.3, 150 mM NaCl, 0.1% BSA, and 0.05% Tween-20] for 1 h at room temperature. A pre-mixture of His-tagged syndecan ectodomains (10 pmole/well) and heparan sulfate at various concentrations was incubated with the VN- or OPN-bound 96-well culture plates for 2 h at room temperature. To detect His-tagged syndecan ectodomains bound to VN or OPN, the plates were washed and exposed to anti-His antibody, HRP-labeled secondary antibody, and substrate. Binding affinity was analyzed by measuring absorbance at 450 nm. Results represent the means ± SD (*n* = 3). The *p* value indicates the comparison between the treatment group and control. ***p* < 0.01.


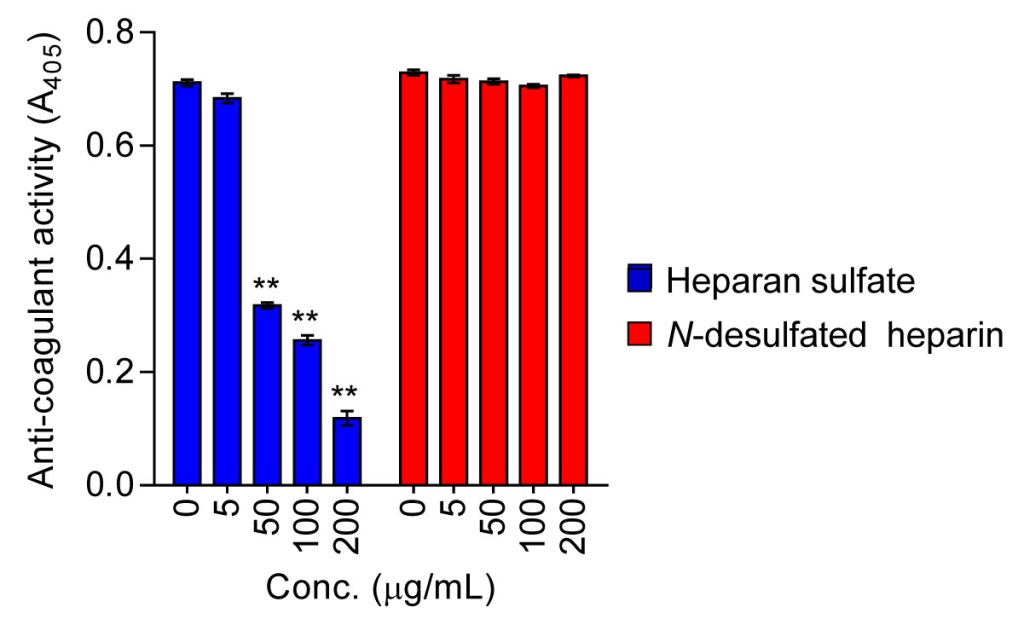


**Supplementary Fig. 6 *N*-desulfated heparin derivatives show no detectable anticoagulant activity.** Heparan sulfate or *N*-desulfated heparin was mixed with anti-thrombin and incubated with factor Xa and its substrate. The activity of factor Xa was quantitatively analyzed by measuring the absorbance at 405 nm using a microplate reader. Results represent the means ± SD (*n* = 3). The *p* value indicates the comparison between the treatment group and control. ***p* < 0.01.


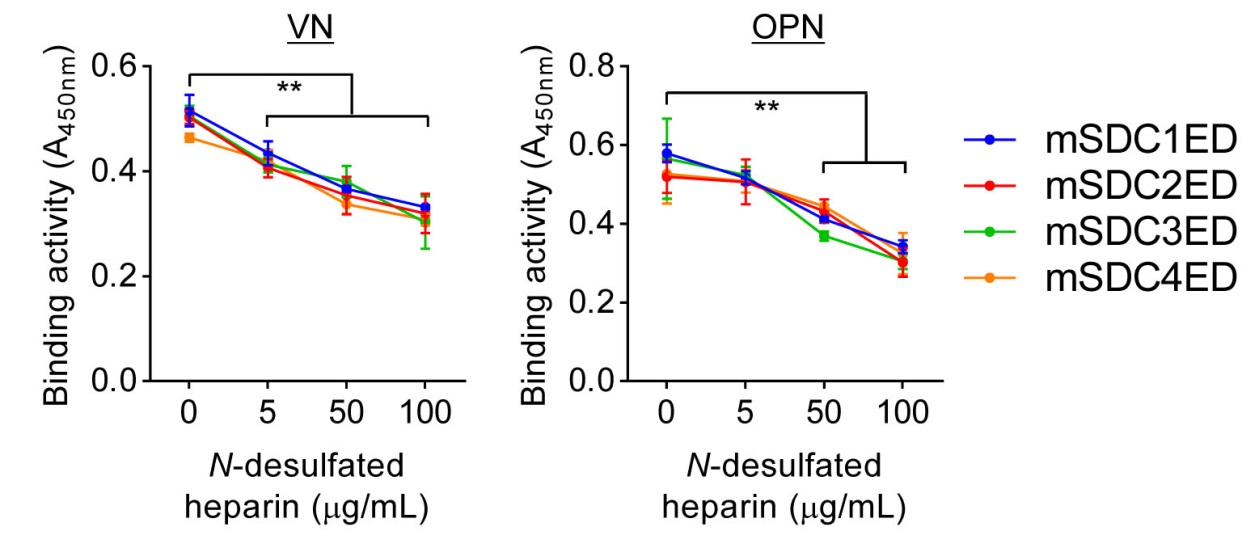


**Supplementary Fig. 7 Binding of *N*-desulfated heparin to VN or OPN.** Binding of *N*-desulfated heparin to VN or OPN was analyzed as described in Supplementary Fig. 5. Results represent the means ± SD (*n* = 3). The *p* value indicates the comparison between the treatment group and control. ***p* < 0.01.

**
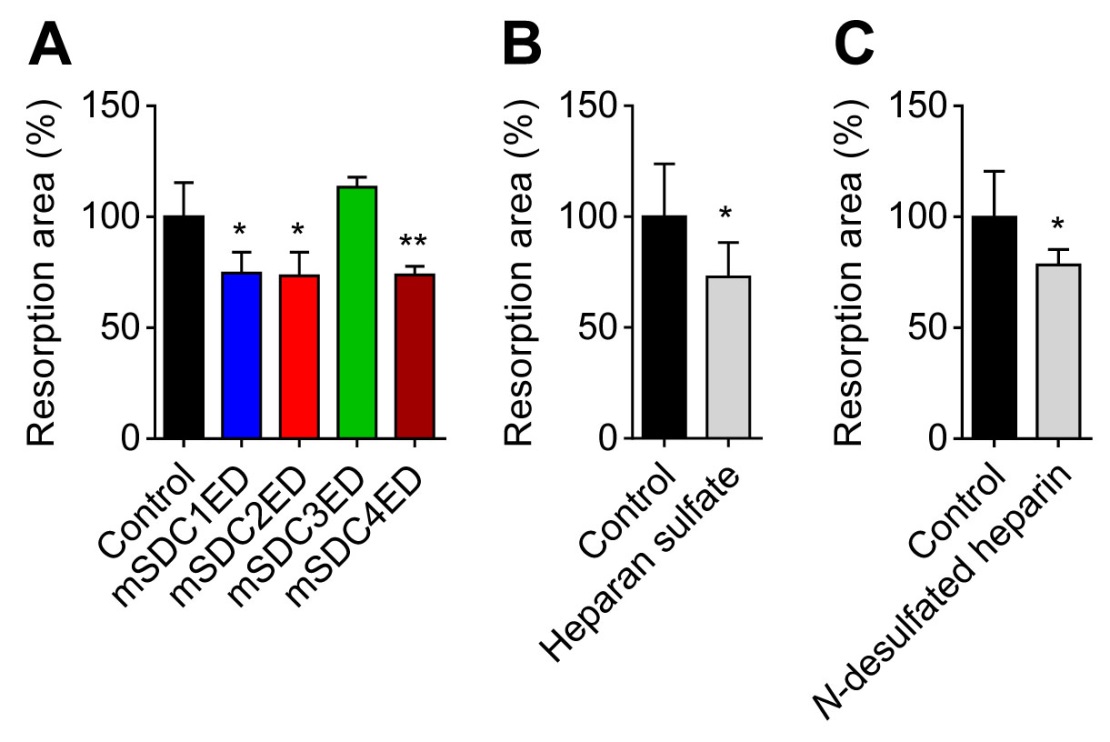
**

**Supplementary Fig. 8 Bone resorption assay *in vitro*.** Mature osteoclasts detached from culture dishes were seeded onto dentine discs in culture medium containing M-CSF (30 ng/mL) and RANKL (100 ng/mL) and further incubated with syndecan-1 to -4 ectodomains (mSDC1ED to mSDC4ED, 1 nM; **a**), heparan sulfate (100 μg/mL; **b**) or *N*-desulfated heparin (100 μg/mL; **c**) for 2 days. The resorbed area of the dentine slices was stained with hematoxylin, analyzed, and expressed as a percentage of the control. Results represent the means ± SD (*n* = 3). The *p* value indicates the comparison between the treatment group and control. **p* < 0.05; ***p* < 0.01.
