# Supplementary material for: Suppressive effect of syndecan ectodomains and N-desulfated heparins on osteoclastogenesis via direct binding to macrophage-colony stimulating factor
Source: Cell Death Dis. 2018 Nov 2;9(11):1119. doi: 10.1038/s41419-018-1167-8 (PMC6215006; doi:10.1038/s41419-018-1167-8)
Supplement: Supplementary file 2 — Supplementary Tables [file 41419_2018_1167_MOESM2_ESM.docx]

**SUPPLEMENTARY INFORMATION**

**Supplementary Tables**

**Supplementary Table 1 Primer lists used in the cloning of syndecan ectodomains expressed in *E. coli*.**

|  | Forward primer (5'-3') | Reverse primer (5'-3') |
| --- | --- | --- |
| Syndecan1 ectodomain | ATGCTAGCTAGCcagcctgccctcccgcaa | GCGCGGCCGCTCAGTGGTGGTGGTGGTGGTGGCTGCCGCCGCCTTCCTTCCTGTCCAAAAGGCTCTG |
| Syndecan2 ectodomain | ATGCTAGCTAGCgagacgagaacagagctg | GCGCGGCCGCTCAGTGGTGGTGGTGGTGGTGGCTGCCGCCGCCTTCTGTCCGTTTAAACAGATTGTC |
| Syndecan3 ectodomain | ATGCTAGCTAGCgctcaacgctggcgcaat | GCGCGGCCGCTCAGTGGTGGTGGTGGTGGTGGCTGCCGCCGCCCTCCTTCCGCTCCAGTATGCTC |
| Syndecan4 ectodomain | ATGCTAGCTAGCgagtcgattcgagagaca | GCGCGGCCGCTCAGTGGTGGTGGTGGTGGTGGCTGCCGCCGCCCTCAGTTCTCTCAAAGATGTTGCTG |

**Supplementary Table 2 Primer lists used in the cloning of syndecan ectodomains expressed in HEK293E mammalian cells.**

|  | Forward primer (5'-3') | Reverse primer (5'-3') |
| --- | --- | --- |
| Syndecan1 ectodomain | GCCAGAATTCATGAGACGCGCGGCGCTCTGC | GCGCGGCCGCTCAGTGGTGGTGGTGGTGGTGGCTGCCGCCGCCTTCCTTCCTGTCCAAAAGGCTCTG |
| Syndecan2 ectodomain | GCCAGAATTCATGCAGCGCGCG TGGATC | GCGCGGCCGCTCAGTGGTGGTGGTGGTGGTGGCTGCCGCCGCCTTCTGTCCGTTTAAACAGATTGTC |
| Syndecan3 ectodomain | GCCAGAATTCATGAAGCCCGGG CCGCCG | GCGCGGCCGCTCAGTGGTGGTGGTGGTGGTGGCTGCCGCCGCCCTCCTTCCGCTCCAGTATGCTC |
| Syndecan4 ectodomain | GCCAGAATTCATGGCGCCTGCC TGCCTGCT | GCGCGGCCGCTCAGTGGTGGTGGTGGTGGTGGCTGCCGCCGCCCTCAGTTCTCTCAAAGATGTTGCTG |
